# Clinical Symptoms of Arboviruses in Mexico

**DOI:** 10.3390/pathogens9110964

**Published:** 2020-11-19

**Authors:** Sushmitha Ananth, Nistha Shrestha, Jesús A. Treviño C., Uyen-sa Nguyen, Ubydul Haque, Aracely Angulo-Molina, Uriel A. Lopez-Lemus, Jailos Lubinda, Rashed Md. Sharif, Rafdzah Ahmad Zaki, Rosa María Sánchez Casas, Diana Cervantes, Rajesh Nandy

**Affiliations:** 1 Department of Biostatistics and Epidemiology, University of North Texas Health Science Center, Fort Worth, TX 76107, USA; sushmitha.ananth@unthsc.edu (S.A.); nistha.shrestha@unthsc.edu (N.S.); Uyen-sa.Nguyen@unthsc.edu (U.-s.N.); Diana.Cervantes@unthsc.edu (D.C.); Rajesh.Nandy@unthsc.edu (R.N.); 2 Department of Urban Affairs at the School of Architecture, Universidad Autónoma de Nuevo León, San Nicolás de los Garza 66455, Nuevo Léon, Mexico; jesus.trevinocn@uanl.edu.mx; 3 Department of Chemical and Biological Sciences/DIFUS, University of Sonora, Hermosillo 83000, Sonora, Mexico; aracely.angulo@unison.mx; 4 Department of Health Sciences, Center for Biodefense and Global Infectious Diseases, Colima 28078, Mexico; ulemus@cebgid.org; 5 School of Geography and Environmental Sciences, Ulster University, Coleraine BT52 1SA, UK; lubinda-j@ulster.ac.uk; 6 Department of Biochemistry, Naogaon Medical College, Naogaon 6500, Bangladesh; naogaonmc@ac.dghs.gov.bd; 7 Centre for Epidemiology and Evidence-Based Practice, Department of Social and Preventive Medicine, University of Malaya, Kuala Lumpur 50603, Malaysia; rafdzah@ummc.edu.my; 8 Entomology Laboratory, Faculty of Veterinary Medicine and Zootechnics, Universidad Autónoma de Nuevo León, General Escobedo 66050, Nuevo Léon, Mexico; sanchezcasasrossy@yahoo.com

**Keywords:** Chikungunya, Dengue, epidemiology, public health, Zika virus

## Abstract

Arboviruses such as Chikungunya (CHIKV), Dengue (DENV), and Zika virus (ZIKV) have emerged as a significant public health concern in Mexico. The existing literature lacks evidence regarding the dispersion of arboviruses, thereby limiting public health policy’s ability to integrate the diagnosis, management, and prevention. This study seeks to reveal the clinical symptoms of CHIK, DENV, and ZIKV by age group, region, sex, and time across Mexico. The confirmed cases of CHIKV, DENV, and ZIKV were compiled from January 2012 to March 2020. Demographic characteristics analyzed significant clinical symptoms of confirmed cases. Multinomial logistic regression was used to assess the association between clinical symptoms and geographical regions. Females and individuals aged 15 and older had higher rates of reported significant symptoms across all three arboviruses. DENV showed a temporal variation of symptoms by regions 3 and 5, whereas ZIKV presented temporal variables in regions 2 and 4. This study revealed unique and overlapping symptoms between CHIKV, DENV, and ZIKV. However, the differentiation of CHIKV, DENV, and ZIKV is difficult, and diagnostic facilities are not available in rural areas. There is a need for adequately trained healthcare staff alongside well-equipped lab facilities, including hematological tests and imaging facilities.

## 1. Introduction

Arboviruses contribute a significant impact on human health among emerging infectious diseases today. The most notable arboviruses currently include Chikungunya (CHIK), Dengue (DENV), and Zika virus (ZIKV). The mosquito vectors known to transmit these arboviruses are *Aedes aegypti* and *Aedes albopictus*. These mosquitoes can be further categorized based on the type of arbovirus transmitted among their varied genus. Globally, the spatial distribution and areas under threat to arboviruses are found in tropical and subtropical regional environments [1]. These regions provide a favorable environment for the survival of *Aedes aegypti* and *Aedes albopictus* mosquitoes, contributing to the prevalence of these mosquitoes worldwide [2,3,4,5].

Clinically, CHIK, DENV, and ZIKV are diagnosed according to signs and symptoms, which pose a challenge when distinguishing these viruses among multiple health systems. This challenge is partly due to their shared clinical manifestations presented by each virus, all based on febrile syndrome [6]. This issue is further complicated by anecdotal triple co-circulation in geographic areas where the vector thrives [6,7,8]. The three arboviruses’ epidemiology somewhat overlaps in regions and often co-circulate through dual or triple co-infections [1,3]. Nonetheless, DENV remains the most prevalent arbovirus, causing disease in the Americas, particularly Central America [9,10].

DENV has been a major public health concern in Mexico since the 1970s [11]. Between 2004 and 2010, Mexico ranked 4th among 30 regions impacted most by the DENV identified by the World Health Organization [12,13] and the 2nd in the Americas after Brazil [13]. To date, Mexico remained a high DENV endemic country and had over a 600% increase in DENV cases from 2001 to 2007 [14]. CHIK and ZIKV have also been detected since then, increasing the probability that these viruses co-circulate. However, the lack of evidence regarding the dispersion of arboviruses in regions through co-circulation [15] and co-infection limits public health policy’s ability to integrate the diagnosis, management, control, and subsequent prevention of these diseases. In 2016, during the peak of ZIKV infections in the Americas, all countries reported or detected DENV and CHIKV outbreaks during the last 15 years were deemed at risk for having outbreaks of ZIKV amidst the panic these three viruses have created in global health circles.

Multiple studies have shown that vector mosquitoes’ environmental suitability led to the observed spread of arboviruses in Mexico [3,5,16,17,18,19,20,21]. However, a few studies have adequately addressed and compared the clinical signs and symptoms of infections transmitted by CHIKV, DENV, and ZIKV [22,23].

This study seeks to reveal the clinical symptom distribution of CHIKV, DENV, and ZIKV, by age group, sex, and region (concerning temporality) across Mexico. The outcomes of this study will inform public health policy and suggest integrated and holistic approaches towards the management of arboviruses in different parts of the country.

## 2. Results

### 2.1. Clinical Symptoms of Each Arbovirus by Age Group and Sex

Between January 2012 and March 2020, de-identified data of 264,736 DENV, 10,394 ZIKV, and 305 CHIKV patients were collected. A total of 264,273 DENV, 10,319 ZIKV, and 305 CHIKV patient’s data were used for analysis. Across all the three arboviruses, most of the patients were female (Table 1). Among the female patients of the arboviruses, 13% of DENV, 14% of CHIKV, and 67% of ZIKV patients were pregnant at the time of infection. The average age of the patients with DENV, ZIKV, and CHIKV was 26, 28, and 33 years, respectively, with the lowest average age found among DENV patients and the highest average among CHIKV patients (Table 1).

Pearson’s Chi-Square test and Fisher’s Exact test yielded significant clinical symptoms for each arbovirus by sex and age group (*p* < 0.05). The reported symptoms for each arbovirus were higher among females compared with the male. Moreover, the reported symptoms were also higher in the age group of 15 years or older. However, in DENV, diaphoresis, photophobia, diarrhea, and sickness were higher among 5–15 years old (Table A1, Table A2, Table A3, Table A4, Table A5 and Table A6).

### 2.2. Clinical Symptoms of Each Arbovirus by Regions in Mexico

In terms of the geographical distribution of the patients, most of the DENV patients were from region 5 (southeast), followed by region 3 (center-west), region 2 (northeast), region 1 (northwest), and region 4 (center). Likewise, for ZIKV patients, most of them were from region 5, followed by region 1, region 3, region 4, and region 2. In addition, for CHIKV patients, most of them were from region 5, followed by region 4, region 2, region 1, and region 3 (Table 1). Detailed information about regions are available in Section 4.3 of Methodology.

**Figure 1:**
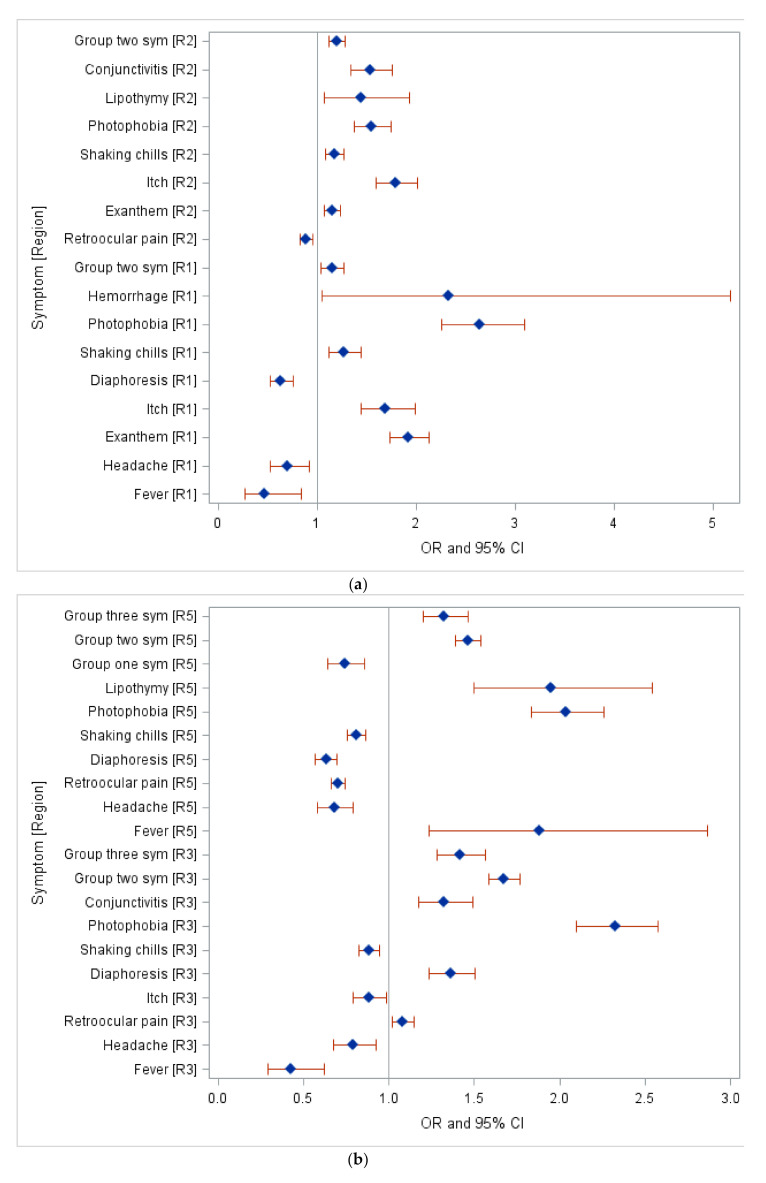
(**a**) Clinical Symptoms of DENV by Northwest Region (R1) and Northeast Region (R2) 2012–2020. Group two symptoms include Sickness, Vomit, Abdominal pain, and Diarrhea. (**b**) Clinical Symptoms of DENV by Center west Region (R3) and Southeast Region (R5) 2012–2020. Group one symptoms include: Myalgias, Arthralgia, Polyarthralgia, and Backpain. Group two symptoms include: Sickness, Vomit, Abdominal pain, and Diarrhea. Group three symptoms include: Nasal congestion, Cough, and Pharyngitis.

**Figure 2:**
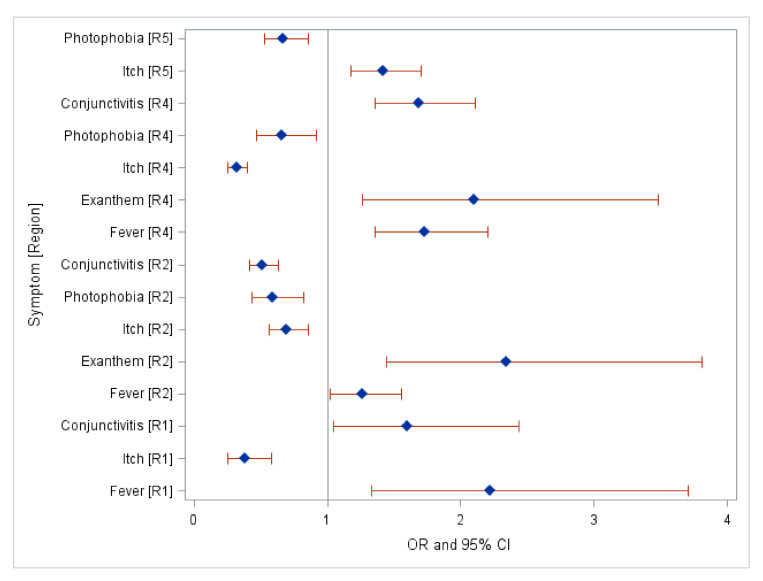
Clinical Symptoms of Zika by Center Region (R4), Northwest Region (R1), Northeast Region (R2), and Southeast Region (R5) 2016–2020.

Based on the multinomial logistic regression (reference group for DENV was region 4, CHIKV was region 2, and for ZIKV it was region 3), most of the symptoms for DENV were found in region 3 and region 5. Additionally, the most prevalent symptoms were photophobia and hemorrhage observed in region 1, itching in region 2, and photophobia in region 3 and 5 (Table A7 and Figure 1a,b). However, no significant clinical symptoms of CHIKV were found across all Mexican Regions. Severe symptoms of DENV were observed prominently in Region 1. This region presented 2.33 times higher odds of severe dengue cases with hemorrhage (95% Confidence Interval (CI):1.05–5.18) and 2.64 times higher odds of photophobia (95% CI: 2.26–3.10) compared to region 4 (Table A7 and Figure 1a,b). Most of the symptoms of ZIKV were observed in region 2 and region 4. Furthermore, fever was the most prevalent symptom in region 1, presenting 2.22 times higher odds of severe cases in region 1 compared to region 3; exanthem presented 2.34 times higher odds of severe cases in region 2 and 2.1 times higher odds of observation in region 4 compared to region 3. Additionally, region 5 characterized itch as a prevalent symptom with 1.42 times higher odds compared to region 3 (Table A8 and Figure 2).

## 3. Discussion

This study identified multiple significant symptoms (*p* < 0.05) by age group, region, sex, DENV, and ZIKV. Our analysis covers eight years, and results indicate that symptoms differ according to sex and age group in the three arboviruses analyzed in this study. Fever, joint pain, myalgia, and skin involvement are common symptoms in all three diseases. However, for CHIKV, polyarthralgia was the only clinical symptom significantly different by sex. For the age group, it was myalgia, vomit, shaking chills, and cough; and there were no significant differences in symptoms by region. The outcomes for CHIKV might be due to insufficient sample size and the fact that CHIKV is a relatively new disease in the country.

Dengue represents 96% of the reported cases (N = 264,267). Dengue fever usually starts with fever and myalgia, but rash and itching are common [24,25]. Headache and retro-orbital pain are more severe than CHIKV and ZIKV fever [24]. Tantawichien [26] revealed in their study that bleeding manifestations are more common in dengue fever, such as epistaxis, gut bleeding, per-vaginal bleeding, per-rectal bleeding. Ascites and pleural effusion are also common in dengue fever due to plasma leakage, causing hypotension and shock leading to death [27]. In Brazil, the most frequently observed symptoms were rash (100%), fever (79.1%), myalgia (74.6%), headache (73.1%), and arthralgia (70.1%) [28] among dengue patients, which is consistent with our findings. On the other hand, patients with ZIKV in Brazil presented skin rash (100%), arthralgia (77.1%), fever and myalgia (74.0%), and non-purulent conjunctivitis (69.8%) as predominant symptoms [28], which is consistent with our findings.

Similar arboviral studies conducted in Cuba and Brazil found pruritus (77.9%), arthralgia (60.0%), headache (50.8%), myalgia (46.1%), fever (34.7%), conjunctivitis (27.9%), with pruritus being the predominant symptom observed across the three arboviral infections. However, in our study, fever is presented as a common symptom in all three arboviral infections [29]. Considering the symptoms of these infections are somewhat overlapping, an avenue for further research could evaluate the timing of the onset of symptoms to support the differential diagnosis of CHIKV, DENV, and ZIKV.

In terms of infection difference by sex, studies have shown that CHIKV, DENV, and ZIKV are predominantly observed among females, consistent with our findings [23]. For instance, studies found that 50% of DENV cases in Mexico were found among females during 2003; however, it decreased by 20% in 2010, and there was no significant difference in 2011 [30]. Chakravarti et al. [31] found that female was more affected and symptoms more prevalent in agreement with our study. However, Kumar et al. [32] reported that males were more affected than females among rural and semi-urban areas. More studies are necessary considering sample size, serological test, case definition, source of the samples, etc. Dengue symptoms among females were related to hemorrhagic findings like thrombocytopenia, anemia, and leucopenia, and they are significantly associated with females compared to males. Severe illness of dengue has been reported as being higher among females. This association may suggest that immune responses may be exacerbated in females as compared to males, with more significant cytokines production and increased permeability in capillaries. This process leads to severe manifestations of dengue in females, in contrast with moderated forms in males. Early changes in hematological markers, including platelets, white blood count, and lymphocyte count, may give additional and important prognostic information [33].

Likewise, for CHIKV and ZIKV studies conducted in 2016 in Mexico, most confirmed cases were among females [23,34]. A high number of arboviruses cases may be seen among females due to seroprevalence. Studies from Mexico have shown that female DENV and ZIKV patients have higher seroprevalence than males [35,36]. However, for CHIKV, one study published in Singapore found that adult males had higher CHIKV seroprevalence than adult females [36]. Fever, rash, joint pain, conjunctivitis, itching, edema may present in ZIKV viral disease. The variability in the number of cases among sexes may be more than just the influence of seroprevalence. Studies have shown that females tend to seek medical assistance more compared to males [37]. Historically, in Latin America, women tend to do most household and water collection duties, which puts them at risk of contracting arboviral diseases due to the proximity to mosquito breeding areas [38].

In terms of infection difference by age group, for all three arboviruses in our study, cases were more common among the older age group (15 years or older), consistent with previous studies [23,34,39]. This phenomenon may be due to the possibility of seropositivity increasing with age; however, the severe case tends to be recognized among the pediatric population [34,39]. For instance, studies published from Mexico in 2006 and 2014 have shown that DENV seroprevalence increases with age [30,35]. Our findings are consistent with Thai et al. [40] and revealed that the old age group developed more dengue symptoms than young adults. Age could be an essential modulator of clinical symptoms, especially for dengue [32]. Another study published in 2017 from Singapore also confirmed that seroprevalence of CHIKV was higher among the 29–70 years age group [41]. Furthermore, Guanche-Garcell et al. [29] found their group with the highest incidence of 40–59 years. A study published from Nicaragua found that ZIKV seropositivity increased with age as well [42].

In terms of infection difference by region, symptomatic infection risks could vary from one region to another. Although the absolute risk of symptomatic infection is related to the virus strain virulence, we should also consider our reliance on patients’ symptoms to understand the disease behavior [40]. Our research found the variability of symptoms by region across Mexico for DENV and ZIKV. The purpose of this research was to understand the clinical symptom distribution across Mexico. The majority of DENV symptoms were found in region 3 and region 5 (Figure 1a,b). A systemic review of DENV regional epidemiology showed that due to regions 3 and 5 being coastal areas in Mexico, they are known to be susceptible to high DENV cases, of which high incidence was seen in Yucatan, Veracruz, and the gulf coast region [30].

Additionally, hemorrhagic symptoms of DENV were observed prominently in Region 1, indicating severe dengue cases. Various hypotheses involving pre-existing dengue antibodies and the virus strain’s origin exist [41,43,44,45]. Many underlying social, economic, and demographic factors may contribute to the severity of dengue cases. Furthermore, serotype surveillance assessment can help shed more light on the distribution of severe cases in Mexico. Various pre-infection factors contribute to the risk of disease severity, including the number of co-circulating serotypes, cross-protective immunity between serotypes, and their pathogenicity [40].

As for ZIKV, most symptoms were found in region 2 and region 4 (Figure 2). Limited studies have shown region 5 to have many cases, but these studies have not examined the cases’ clinical symptoms as our research did [23,46]. The outcomes from the regional variation of symptoms may be used to look at the severity of symptoms comparing endemic vs. non-endemic municipalities to narrow down other regional differences, healthcare worker practices, and the possible role of access to publicly funded healthcare services. For instance, a study conducted in 2002 showed that Mexico’s state-level indicators had larger disparities in access to healthcare services that are publicly funded. This, in turn, may influence a person’s decision to get treatment in the early stages of their infection [44].

Additionally, in 2018, in Hidalgo (Mexico), a study was conducted to assess risk perception and knowledge of diseases transmitted by *Ae. aegypti* among healthcare workers. The nurses and vector operating staff had the lowest level of expertise. This study’s outcome showed the potential for variation in healthcare workers’ knowledge, practices, and attitudes [47,48]. Further studies may investigate the connection between the severity of the arboviral disease’s clinical symptoms and comorbidities by demography and geographical variables. Future research could also concentrate on assessing the knowledge, practices, and challenges healthcare professionals face in regions where a high number of symptoms were found to ensure proper diagnosis and treatment.

## 4. Methodology

### 4.1. Arbovirus Cases and Diagnosis

The State Public Health Laboratories of Mexico identify CHIKV, DENV, and ZIKV. Confirmed cases are reported to the local facility within 24 h of detection. This information is then relayed to the General Directorate of Epidemiology, responsible for collecting the data at the national level [49]. We assessed Mexico’s national data of arboviral infections, including information from 2511 municipalities from January 2012 to March 2020.

De-identified daily case records from January 2012 to March 2020 of CHIKV, DENV, and ZIKV were obtained at the municipality level from the Mexican Ministry of Health. The clinical data included information about confirmed diagnosis categorized into severe or mild, date of symptoms onset, and diagnostic methods.

### 4.2. Case Definition, Infection, and Diagnostic Tools

All CHIKV, DENV, and ZIKV cases are lab-confirmed. Dengue infection was determined through the detection of DENV NS1 antigen using the PanBio Dengue NS1 Early ELISA (Inverness Medical Innovations), following the manufacturer’s instructions, which is widely used for the early detection of DENV infection [50]. In addition, at the States Laboratories of Public Health of Mexico, negative samples were subjected to serological analyses using the Enzyme-Linked Immunosorbent Assay (ELISA) to detect Immunoglobulin M (IgM). Immunoglobulin G (IgG) using the Dengue IgM/IgG Capture ELISA Tests (PanBio, Brisbane, Queensland, Australia), following the manufacturer’s protocols, were also utilized at the States Laboratories of Public Health of Mexico [50]. DENV infection was confirmed and recorded at the Institute for Epidemiological Diagnosis and Reference (InDRE), of the Ministry of Health, following the Mexican guidelines for dengue surveillance in Mexico [50]. All clinical cases were identified at the municipality level based on the hospital report form’s address. Monthly average DENV cases were aggregated for each municipality.

**Figure 3:**
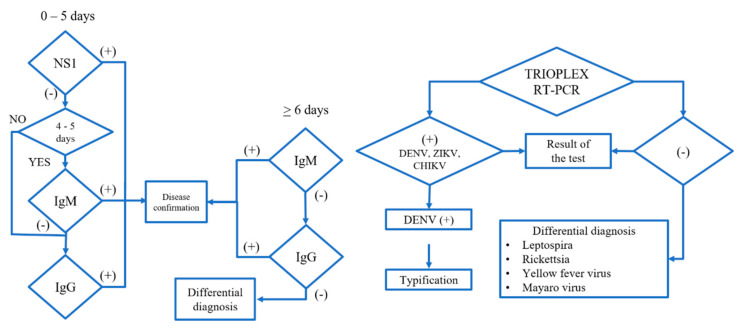
The general algorithm for detecting arboviruses and differential diagnosis of febrile diseases. Adapted from the Institute of Epidemiological Diagnosis and Reference, Ministry of Health, Mexico, 2019 [51].

CHIKV and ZIVV cases in acute serum samples (0–5 days) were determined and confirmed using the Center for Disease Control (CDC) Trioplex Real-time (RT-PCR) Assay (Figure 3), following manufacturer’s protocol at InDRE [33,49,50]. Negative samples were subjected to differential diagnosis for Leptospira, Rickettsia, Yellow Fever virus, and Mayaro virus. Collection of samples, transportation, and confirmation at InDRE were carried out following the Mexican guidelines for arboviral diseases laboratory-based surveillance in the Mexican territory [33,51].

### 4.3. Statistical Analysis

Statistical analysis was performed to determine the symptoms by age and sex. Pearson’s Chi-Square test and Fisher’s Exact Test was performed to identify significant clinical symptoms by sex and age group (0–4, 5–15, greater than 15) for each disease. A multinomial logistic regression (region = dependent and symptoms = independent variables) was performed to determine the arboviruses’ significant clinical symptoms across Mexico.

**Figure 4:**
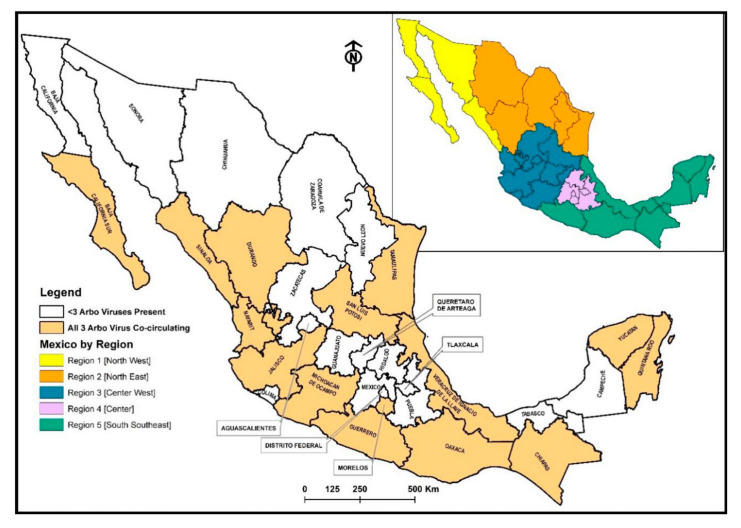
Co-circulation Map of CHIKV, DENV, and ZIKV in Mexico and Map of Mexico by Region, (region 1 (north west): Baja California, Baja California Sur, Sonora, and Sinaloa; region 2 (north east): Durango, Coahuila, Nuevo Leon, and Tamaulipas; region 3 (center west): Zacatecas, San Luis Potosi, Aguascalientes, Guanajuato, Queretaro, Nayarit, Jalisco, Colima and Michoacán; region 4 (center): Hidalgo, Mexico city, Distrito Federal, Morelos, Puebla, and Tlaxcala; and region 5 (south southeast): Oaxaca, Guerrero, Veracruz, Chiapas, Tabasco, Campeche, Quintana Roo, and Yucatan [52].

For the multinomial logistic regression test, (only significant symptoms in bivariate analysis were grouped) symptoms were grouped (group one: myalgias, arthralgia, polyarthralgia, and backpain; group two: sickness, vomit, abdominal pain, and diarrhea; and group three: nasal congestion, cough, and pharyngitis) into broader categories. Mexico was divided into five regions based on Mexico geography and economic development as outlined by Contreras, Cabanas, and Nuno-(region 1 (north west): Baja California, Baja California Sur, Sonora, and Sinaloa; region 2 (north east): Durango, Coahuila, Nuevo Leon, and Tamaulipas; region 3 (center west): Zacatecas, San Luis Potosi, Aguascalientes, Guanajuato, Queretaro, Nayarit, Jalisco, Colima, and Michoacán; region 4 (center): Hidalgo, Mexico City, Distrito Federal, Morelos, Puebla, and Tlaxcala; and region 5 (south southeast): Oaxaca, Guerrero, Veracruz, Chiapas, Tabasco, Campeche, Quintana Roo, and Yucatan, Figure 4) [52]. As for reference for the test, region 4 served as the reference for DENV, region 2 for CHKV, and region 3 for ZIKV. The regions’ references were selected based on a frequency test, where regions with the lowest number of confirmed CHIKV/DENV/ZIKV were selected. SAS 9.4 was used to conduct the analysis.

## 5. Study Limitations

The study has potential limitations. There is a lack of genetic analyses of the positive samples to determine if emergent genetic variations among the included viruses could lead to a severe or mild clinical course, especially during viral co-infections or second infections. Some reports [53,54,55,56] have shown that some mutations in the DENV genome lead to a more severe progression of the dengue disease in tropical regions. Moreover, there is an unpredictable clinical course in dengue serotypes among coinfected patients or coinfected with other flaviviruses. Undoubtedly, determining the genetic variability would enable us to deploy preventive medical strategies against arboviruses in endemic areas, especially in vulnerable regions where diagnostic and clinical capabilities are minimal.

The regional variation in clinical symptoms may be biased by local economic development and pre-existing diseases. The data contains information on the symptoms only for patients confirmed by clinical and laboratory diagnoses with the three arboviruses we considered. This study did not view any tested negative samples and did not predict the positive or negative cases based on clinical signs and symptoms. Instead, the goal was to study each disease and the relationship between the reported symptoms with geographic regions. However, a knowledge of such a relationship may inform a physician better regarding regional variation in symptoms among patients. It may lead to a better diagnosis, especially in developing nations, where there is not enough facility and laboratory diagnosis resources.

## 6. Conclusions

This study adds valuable findings to the current researchers’ quest to distinguish the overlapping symptoms between CHIKV, DENV, and ZIKV. This study also found significant clinical symptoms for these three arboviruses by age group, sex, and regions across Mexico. Regions with some majority of symptoms show that there is potential to re-direct resources, assess healthcare workers’ knowledge, perceive current prevention and management practices, tailor public health messaging, and increase women’s inclusion in implementing vector control measures. The outcomes of this study will inform public health policy and suggest integrated and holistic approaches towards the management of arboviruses in different parts of Mexico and elsewhere.

## Figures and Tables

**Table 1:** Demographic information of arboviruses patients.

	Dengue (N = 264,273)	Chikungunya (N = 305)	Zika (N = 10,319)
**Sex N (%)**			
Female	145,878 (55)	186 (61)	154,809 (56)
Male	118,389 (45)	119 (39)	120,082 (44)
Age in years Mean (SD)	26 (18.7)	33 (19.1)	28 (12.4)
Pregnancy N (%)			
Yes	5696 (13.30)	10 (13.5)	5476 (67)
No	37,137 (86.7)	64 (86.5)	2691 (33)
Region N (%)			
1 (North west)	26,921 (10)	26 (9)	710 (7)
2 (North east)	28,894 (11)	7 (2) [Ref] *	1215 (12)
3 (Center west)	50,779 (20)	23 (8)	639 (6) [Ref] *
4 (Center)	22,324 (9) [Ref] *	11 (4)	2110 (21)
5 (South east)	129,976 (50)	235 (78)	5481 (54)

Ref * = Reference.

## Appendix: Tables A1–A8

**Table A1:** Clinical symptoms from confirmed DENV cases by sex from 2012 to 2020.

Symptoms	Total (N = 264,267)		Female (N = 145,878)		Male (N = 118,389)		*p*-Value *
	Symptomatic Cases	Cases with Missing Symptoms	Symptomatic Cases	%	Symptomatic Cases	%	
Fever	263,837	10	145,611	55.19	118,227	44.81	0.003
Myalgias	244,636	397	135,666	55.46	108,971	44.54	<0.0001
Arthralgias	231,277	407	128,508	55.56	102,769	44.44	<0.0001
Abdominal Pain	32,430	57,649	18,413	56.78	14,017	43.22	<0.0001
Polyarthralgias	27,315	155,201	16,137	59.08	11,179	40.92	<0.0001
Backache	18,372	182,327	10,638	57.9	7734	42.1	0.0001
Photophobia	11,486	182,328	6825	59.42	4661	40.58	<0.0001
Diarrhea	7442	182,321	3750	50.39	3692	49.61	<0.0001
Conjunctivitis	5935	182,319	3169	53.4	2766	46.6	<0.0001
Cough	4675	182324	2457	52.56	2218	47.44	<0.0001
Pharyngitis	4956	182,323	2725	54.98	2231	45.02	0.014
Sickness	48,245	182,320	27,984	58	20,261	42	<0.0001
Headache	253,725	366	140,751	55.47	112,975	44.53	<0.0001
Itch	6396	224,855	3938	61.57	2458	38.42	<0.0001
Vomit	42,869	76,174	24,289	56.66	18,580	43.34	<0.0001
Retroocular pain	174,348	7044	96,723	55.48	77,625	44.52	<0.0001
Exanthem	77,140	84	43,723	56.68	33,418	43.32	<0.0001

* *p*-value based on Pearson’s Chi-Square test.

**Table A2:** Clinical symptoms from confirmed DENV cases by age group from 2012 to 2020.

Symptoms	Cases with Missing Symptoms	Age Group (0–4) (N = 25,895)		Age Group (5–15) (N = 72,854)		Age Group (>15) (N = 165,523)		*p*-Value *
		Symptomatic Cases	%	Symptomatic Cases	%	Symptomatic Cases	%	
Fever	10	25,765	9.77	72,676	27.55	165,396	62.69	<0.0001
Myalgias	397	22,057	9.02	65,478	26.77	157,101	64.22	<0.0001
Arthralgias	407	19,775	8.55	59,946	25.92	151,555	65.53	<0.0001
Abdominal Pain	57,649	4270	13.17	10,564	32.57	17,596	54.26	<0.0001
Polyarthralgias	155,201	2079	7.61	6098	22.32	19,138	70.06	<0.0001
Backache	182,327	4215	22.94	6649	36.19	7508	40.87	<0.0001
Diaphoresis	182,326	2559	26.23	3827	39.23	3369	34.54	<0.0001
Shaking chills	182,321	5689	22.76	9532	38.13	9779	39.12	<0.0001
Photophobia	182,328	2788	24.27	4460	38.83	4238	36.9	<0.0001
Diarrhea	182,321	1896	25.48	2987	40.14	2559	34.39	0.0007
Conjunctivitis	182,319	1122	18.9	2071	34.89	2742	46.2	<0.0001
Nasal congestion	182,323	719	22	1249	38.22	1249	39.78	<0.0001
Pharyngitis	182,323	1122	22.64	1882	37.97	1952	39.39	<0.0001
Sickness	182,320	11,870	24.6	20,318	42.11	16,057	33.28	<0.0001
Headache	366	23,135	9.12	69,713	27.48	160,877	63.41	<0.0001
Itch	224,855	220	3.44	1233	19.28	4943	77.28	<0.0001
Vomit	76174	8004	18.67	16,372	38.19	18,493	43.14	<0.0001
Retroocular pain	7044	15,868	9.1	47,189	27.07	111,291	63.83	<0.0001
Exanthem	84	7660	9.93	21,512	27.89	47,968	62.18	0.0072

* *p*-value based on Pearson’s Chi-Square test.

**Table A3:** Clinical symptoms from confirmed CHIK cases by sex from 2014 to 2019.

Symptoms	Total (N = 305)		Female (N = 186)		Male (N = 119)		*p*-Value *
	Symptomatic Cases	Cases with Missing Symptom	Symptomatic Cases	%	Symptomatic Cases	%	
Polyarthralgias	100	102	70	70	30	30	0.04

* *p*-value based on Pearson’s Chi-Square test.

**Table A4:** Clinical symptoms from confirmed CHIK cases by age group from 2014 to 2019.

Symptoms	Cases with Missing Symptoms	Age Group (0–4) (N = 8)		Age Group (5–15) (N = 52)		Age Group (>15) (N = 245)		*p*-Value *
		Symptomatic Cases	%	Symptomatic Cases	%	Symptomatic Cases	%	
Myalgias	0	6	2.2	42	15.38	225	82.42	0.024
Vomit	9	1	2.56	12	30.77	26	66.67	0.037
Shaking chills	37	0	0	14	12.96	94	87.04	0.033 (F)
Cough	37	2	7.41	1	3.7	24	88.89	0.049 (F)

* *p*-value either based on Pearson’s Chi-Square test or Fisher’s Exact test (F).

**Table A5:** Clinical symptoms from confirmed ZIKV cases by sex from 2016 to 2020.

Symptoms	Total (N = 10,319)		Female (N = 8745)		Male (N = 1574)		*p*-Value *
	Symptomatic Cases	Cases with Missing Symptom	Symptomatic Cases	%	Symptomatic Cases	%	
Fever	7226	0	5810	80.4	1416	19.6	<0.0001
Myalgias	7739	13	6417	82.92	1322	17.08	<0.0001
Arthralgias	6659	25	5511	82.76	1148	17.24	<0.0001
Retroocular pain	4967	40	4043	81.4	924	18.6	<0.0001
Exanthem	9728	9	8333	85.66	1395	14.34	<0.0001
Abdominal Pain	951	69	772	81.18	179	18.82	0.001
Polyarthralgias	674	63	516	76.56	158	23.44	<0.0001
Diaphoresis	688	157	528	76.74	160	23.26	<0.0001
Shaking chills	2145	57	1609	75.01	536	24.99	<0.0001
Photophobia	1350	77	1103	81.7	247	18.3	0.0006
Diarrhea	700	70	536	76.57	164	23.43	<0.0001
Conjunctivitis	5033	37	4230	84.05	803	15.95	0.043
Nasal congestion	602	75	485	80.56	117	19.44	0.003
Cough	498	68	391	78.51	107	21.49	<0.0001
Pharyngitis	1096	70	862	78.65	234	21.35	<0.0001
Headache	8408	10	7013	83.41	1395	16.59	<0.0001
Itch	6751	42	5859	86.79	892	13.21	<0.0001

* *p*-value based on Pearson’s Chi-Square test.

**Table A6:** Clinical symptoms from confirmed ZIKV cases by age group from 2016 to 2020.

Symptoms	Cases with Missing Symptoms	Age Group (0–4) (N = 144)		Age Group (5–15) (N = 747)		Age Group (> 15) (N = 9428)		*p*-Value *
		Symptomatic Cases	%	Symptomatic Cases	%	Symptomatic Cases	%	
Fever	0	126	1.74	606	8.39	6494	89.87	<0.0001
Myalgias	13	74	0.96	558	7.21	7107	91.83	<0.0001
Arthralgias	25	56	0.84	440	6.61	6163	92.55	<0.0001
Retroocular pain	40	43	0.87	383	7.71	4541	91.42	<0.0001
Exanthem	9	123	1.26	679	6.98	8926	91.76	<0.0001
Vomit	68	24	2.37	86	8.48	904	89.15	0.004
Backpain	90	9	0.34	150	5.61	2516	94.06	<0.0001
Conjunctivitis	37	60	1.19	310	6.16	4663	92.65	<0.0001
Sickness	53	24	0.91	186	7.09	2415	92	0.05
Headache	10	86	1.02	656	7.8	7666	91.18	<0.0001
Itch	42	72	1.07	435	6.44	6244	92.49	<0.0001

* *p*-value based on Pearson’s Chi-Square test.

**Table A7:** Clinical symptoms of DENV in Mexico by region 2012–2020.

Clinical Symptom	Odds Ratio Estimate	Lower CL	Upper CL
Fever (R*1)	0.47	0.27	0.84
Headache (R1)	0.70	0.53	0.92
Exanthem (R1)	1.93	1.74	2.14
Itch (R1)	1.69	1.44	1.99
Diaphoresis (R1)	0.63	0.53	0.75
Shaking chills (R1)	1.27	1.12	1.44
Photophobia (R1)	2.64	2.26	3.10
Hemorrhage (R1)	2.33	1.05	5.18
Group two sym (R1)	1.15	1.04	1.28
Retroocular pain (R2)	0.89	0.83	0.95
Exanthem (R2)	1.15	1.07	1.23
Itch (R2)	1.79	1.59	2.02
Shaking chills (R2)	1.17	1.08	1.27
Photophobia (R2)	1.55	1.38	1.74
Lipothymy (R2)	1.44	1.07	1.93
Conjunctivitis (R2)	1.54	1.35	1.76
Group two sym (R2)	1.20	1.12	1.28
Fever (R3)	0.42	0.29	0.62
Headache (R3)	0.79	0.67	0.93
Retroocular pain (R3)	1.08	1.02	1.15
Itch (R3)	0.88	0.79	0.99
Diaphoresis (R3)	1.36	1.23	1.50
Shaking chills (R3)	0.88	0.82	0.95
Photophobia (R3)	2.32	2.10	2.58
Conjunctivitis (R3)	1.32	1.17	1.49
Group two sym (R3)	1.67	1.58	1.76
Group three sym (R3)	1.42	1.28	1.57
Fever (R5)	1.88	1.24	2.87
Headache (R5)	0.68	0.58	0.79
Retroocular pain (R5)	0.70	0.66	0.75
Diaphoresis (R5)	0.63	0.57	0.70
Shaking chills (R5)	0.81	0.75	0.87
Photophobia (R5)	2.04	1.83	2.26
Lipothymy (R5)	1.95	1.5	2.54
Group one sym (R5)	0.75	0.64	0.86
Group two sym (R5)	1.46	1.39	1.54
Group three sym (R5)	1.33	1.20	1.47

* R = region and number are associated with region number.

**Table A8:** Clinical symptoms of ZIKV in Mexico by region 2016–2020.

Clinical Symptom	Odds Ratio Estimate	Lower CL	Upper CL
Fever (R*1)	2.22	1.33	3.71
Itch (R1)	0.38	0.25	0.58
Conjunctivitis (R1)	1.6	1.05	2.44
Fever (R2)	1.26	1.02	1.56
Exanthem (R2)	2.34	1.44	3.81
Itch (R2)	0.69	0.56	0.86
Photophobia (R2)	0.59	0.43	0.82
Conjunctivitis (R2)	0.51	0.42	0.63
Fever (R4)	1.73	1.36	2.2
Exanthem (R4)	2.1	1.26	3.48
Itch (R4)	0.32	0.25	0.4
Photophobia (R4)	0.66	0.47	0.92
Conjunctivitis (R4)	1.69	1.36	2.11
Itch (R5)	1.42	1.18	1.7
Photophobia (R5)	0.67	0.53	0.86

* R = region and number are associated with region number.
